# China’s drug clinical trial institution record-keeping system: Qualification requirements for PI are the key

**DOI:** 10.3389/fphar.2023.1052977

**Published:** 2023-03-02

**Authors:** Nanqu Huang, Wendi Huang, Yong Luo, Juan Huang

**Affiliations:** ^1^ National Drug Clinical Trial Institution, Third Affiliated Hospital of Zunyi Medical University (The First People’s Hospital of Zunyi), Zunyi, Guizhou, China; ^2^ Key Laboratory of Basic Pharmacology and Joint International Research Laboratory of Ethnomedicine of Ministry of Education, Zunyi Medical University, Zunyi, Guizhou, China; ^3^ School of Public Health, Zunyi Medical University, Zunyi, Guizhou, China

**Keywords:** drug clinical trial institution, principal investigator, record-keeping system, clinical trial management, clinical trial

## Abstract

It has been 3 years since China implemented new management regulations for drug clinical trial institutions in December 2019, the most important of which is to change the qualification recognition of drug clinical trial institutions into record-keeping system. The original intention of the institution record-keeping system was to solve the shortage of clinical trial resources in China, effectively expand the number of clinical trial institutions, and effectively alleviate the contradiction between medical treatment and scientific research. After implementing the record-keeping system, although these goals have been achieved to a certain extent, there are still areas worthy of optimization and improvement. Therefore, we evaluated the new process, in particular the requirements, in order to see what possible barriers in the record-keeping system of institutions. We find that the requirements for principal investigator (PI) qualifications are the key to the record-keeping system. This reflects the shift of Chinese regulators’ supervision of clinical trials to supervision of the ability to conduct clinical trials. However, the ambiguity of the definition of PI qualification has hindered implementation of the record-keeping system and reduced the release of clinical trial resources.

## Introduction

On 1 December 2019, the new Drug Administration Law of China was officially implemented (NMPA and NHC). This is the first version of China’s regulatory document that officially guides clinical trials since China joined the International Council for Harmonization (ICH) in 2017, and it has attracted much attention. The law has made important changes to clinical trials compared to the previous. First, the approval of clinical trials has been changed to an implied licensing—Article 19; second, the management of clinical trial institution has been changed to a record-keeping system—Article 19; third, the importance of ethics has been emphasized—Article 20, 21, 22; last, extended clinical trials have been supported—Article 23 (S.C.o.t.N.P.s. Congress, 2019). In particular, some new regulations have been instituted on the qualification requirements for principal investigators (PIs) (NMPA, 2019a). Regulators hope to solve the shortage of clinical trial resources in China by optimizing the process. And by emphasizing PI requirements in the record-keeping system of institutions, in order to ensure the quality of clinical trials. Because the PI plays a key role in the project (O'Kane et al., 2020), regulators emphasize the PI’s qualifications as a positive. It is worthy of recognition that the regulatory authorities emphasize the qualifications of PIs. The high requirement for PIs is an important means to ensure the quality of the project. Therefore, we evaluated the new process, in particular the requirements, in order to see what possible barriers in the record-keeping system of institutions. Three years after the implementation of the new law, we can review China’s supervision of clinical trials from the perspective of institutional filing.

## From qualification recognition to record-keeping system

**FIGURE 1:**
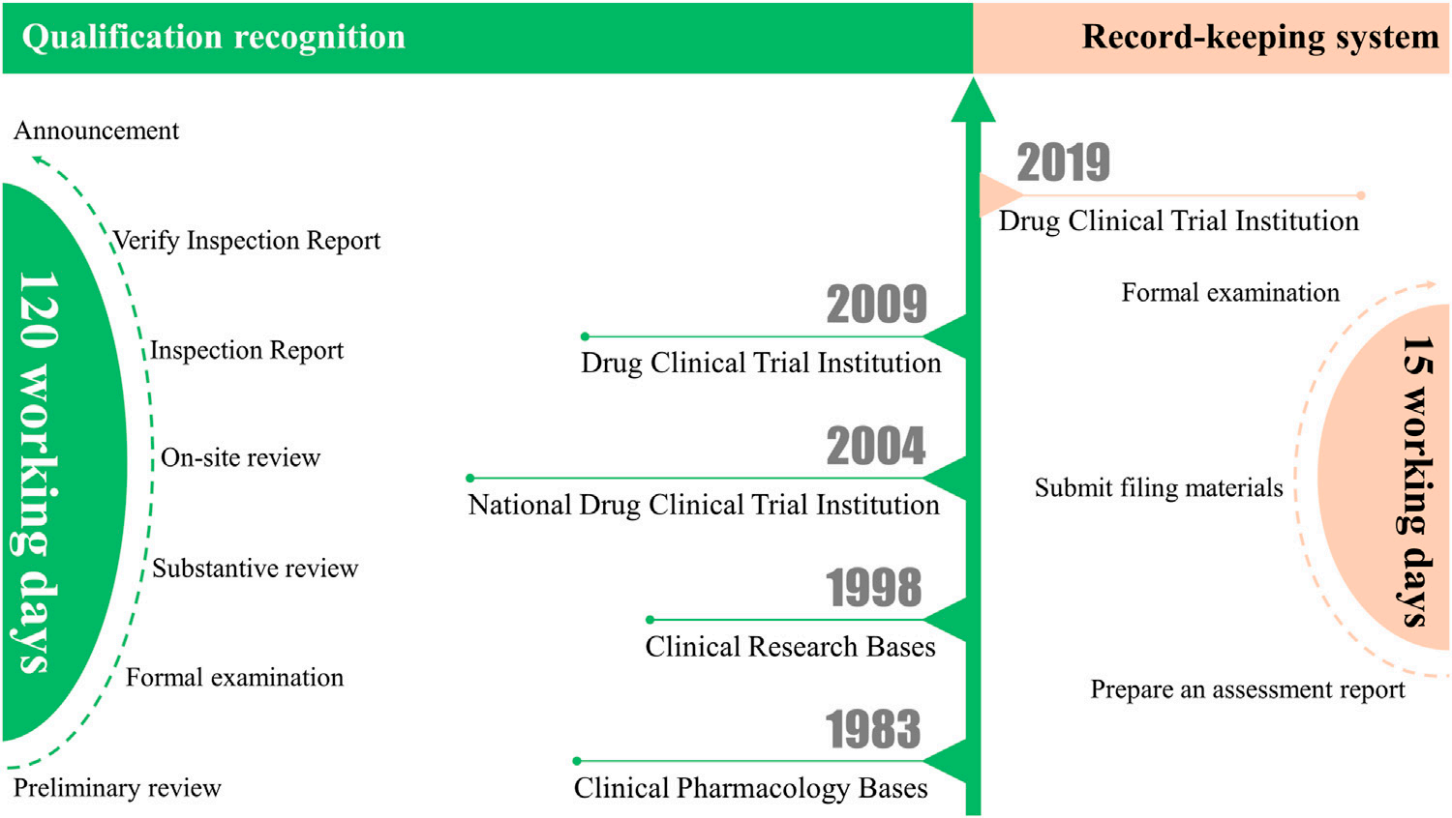
From qualification recognition to record-keeping system: Differences between the clinical trial institution qualification recognition and the record-keeping system.

Before the record-keeping system, China’s clinical trial institutions went through a qualification accreditation model for many years (SFDA, 2004). The predecessor of China’s drug clinical trial institutions was the clinical pharmacology base. In 1983, the Ministry of Health authorized 14 medical institutions with strong comprehensive strength to become the first clinical pharmacology bases (Yang et al., 2021). In 1998, the Ministry of Health of China promulgated the first version of the “Good Clinical Practice Guidelines (for Trial Implementation)”, which reviewed and inspected the previously identified clinical pharmacology bases in accordance with the requirements of good clinical practice and renamed them “clinical research bases” (MOH, 1998; Chen et al., 2018). In 2004, the State Food and Drug Administration (SFDA) and the Ministry of Health jointly promulgated the “Measures for the Accreditation of Drug Clinical Trial Institutions (for Trial Implementation)”. The accredited clinical research base was officially renamed the “National Drug Clinical Trial Institution” (SFDA, 2004). In 2009, the SFDA issued the “Standards Bulletin for Certification of Qualifications of Drug Clinical Trial Institutions” (SFDA, 2009), which initiated institutional qualification work. However, on 22 July 2015, because the clinical trial data were widely questioned, the China Food and Drug Administration (CFDA) issued the “Announcement of the China Food and Drug Administration on Carrying out Self-checking and Checking of Drug Clinical Trial Data (No. 117, 2015)” (CFDA, 2015). This is a famous event in the field of clinical trials in China, also known as the “722” event (Woodhead, 2016). Subsequently, enthusiasm for conducting drug clinical trials waned. Seven years later, it is still embarrassing to look back on the large-scale withdrawal of clinical trial declarations during that period. However, with the huge demand for the development of biomedicine in China, the number of clinical trial institutions that can conduct clinical trials is seriously insufficient. The voice of the demanding lower entry requirements and simplified application procedures is increasing day by day. Therefore, on 29 November 2019, the National Medical Products Administration (NMPA) and the National Health Commission jointly issued the “Administrative Regulations on Drug Clinical Trial Institutions” (NMPA, 2019b) (Figure 1).

## Changes in the application process and actual effects

Comparing the difference between the clinical trial institution qualification recognition and the record-keeping system, we can see that the application process has changed as follows: 1. There is no need to report application materials level by level, which simplifies the application process. 2. There is no need for on-site inspection; only the form of application materials needs to meet the requirements (Figure 1). Overall, the record-keeping system greatly simplifies the application process and shortens the application time compared to qualification recognition. Under the qualification recognition, it will take at least 120 working days from the initial submission of application materials to obtain the qualification recognition issued by the CFDA. However, under the record-keeping system, drug clinical trial institutions only need to register and submit materials on the drug clinical trial institution record-keeping management information platform (https://beian.cfdi.org.cn/CTMDS/apps/pub/drugPublic.jsp). The statutory time from online submission of materials to completion of record-keeping can be accomplished within 15 working days. Other than that, the institutional access conditions have not changed much. Only for Phase I clinical trials of new drugs or clinical trials of drugs with higher clinical risks have higher requirements for the qualifications of medical institutions, requiring hospitals to be tertiary medical institutions. It also specifically stipulates the conditions for the Centers for Disease Control and Prevention (CDC) as a drug clinical trial institution.

**FIGURE 2:**
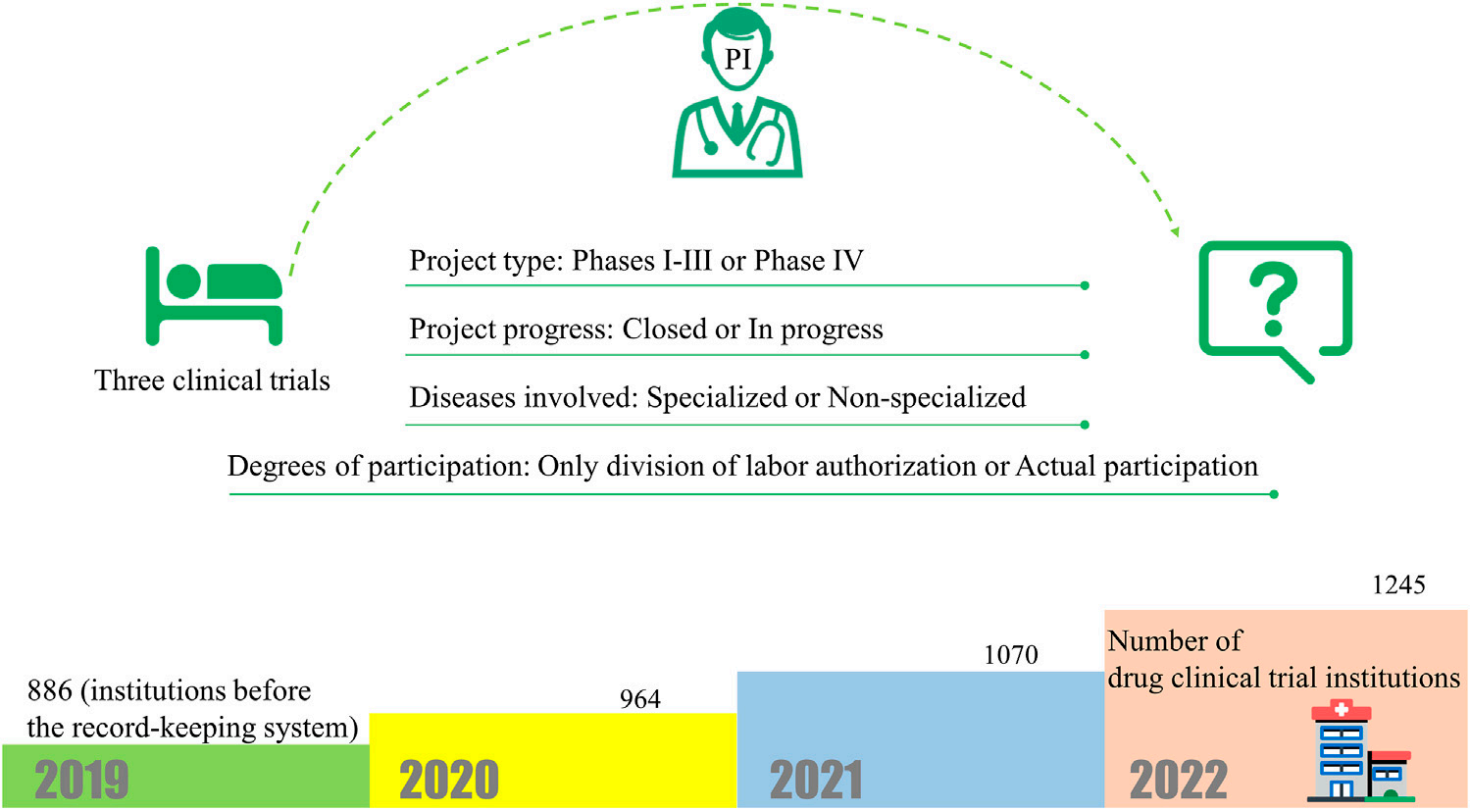
The new regulations require that PIs have participated in more than three clinical trials, but the regulations do not explain much about the types of clinical trials. And changes in the number of clinical trial institutions after the implementation of the record-keeping system.

The arrival of the record-keeping system has brought about an increase in the number of Chinese clinical trial institutions. As of 5 September 2022, after 3 years of the record-keeping system (Figure 2), the number of clinical trial institutions that have obtained qualifications has increased by 371 to 1,257 (41.87% increase compared to 2019) (NMPA, 2022). Of course, we can also find interesting phenomena here. For example, some provincial Centers for Disease Control and Prevention (CDC) have registered many researchers from grass-roots CDCs, but they do not actually belong to the same unit geographically. At the same time, there is the case of the Shanghai CDC, where a PI in the record-keeping system belongs to several geographically different branches. There are also temporary COVID-19 specialty hospitals, such as Wuhan Huoshenshan Hospital (this hospital does not have an actual grade; it is displayed as “other” in the record-keeping system). Because temporary hospitals such as Huoshenshan hospital are easy to classify, we can see that the main reason that grass-roots hospitals find it difficult to file is related to the stringent requirements for PIs.

## Qualification requirements for PI are the key

The new regulations require that PIs have participated in more than three clinical trials, although the “Measures for the Administration of Drug Registration” stipulates that clinical drug trials refer to drug research conducted with humans for the purpose of determining drug safety and effectiveness. However, the regulations do not explain much about the types of clinical trials. This has created a series of difficult questions to answer. For example, does a PI’s clinical trial only need to be authorized by the division of labour, or does it need to actually participate? Do the projects that the PI participated in need to be closed? Is it feasible to authorize participation in non-specialized clinical trial projects, such as ophthalmology researchers participating in respiratory projects?

This also provides flexible operation space for the drug administration department of each province. After the new clinical trial institution is filed, the provincial bureau will inspect it within 60 days. The standards and rules are in the hands of the provincial bureaus. The standards of each province are different, and many provinces have not made the standards clear. The most stringent requirements, the experience of three registered drug studies as a subcentre PI, are generally met in Phases I-III in cities such as Beijing (B.M.D. Administration, 2021). Some provinces have also relaxed the regulations to include Phase IV clinical trials projects, or as sub-I in sub-centres. It is this requirement for PIs that has prevented the release of some clinical trial resources. In China, due to the dominance of public hospitals, most doctors work in one hospital for a long time, and staff mobility is poor. Therefore, in hospitals that are not qualified to conduct drug clinical trials, this seemingly simple condition is indeed difficult to achieve, especially for some low-level hospitals. As a result, the solution to this problem becomes complicated and strange, trapped in a chicken-and-egg cycle. In qualified hospitals, the PI is often used to obtain authorization for the institution’s other departments to participate in clinical trial projects to meet this requirement. In unqualified hospitals, meeting this requirement means recruiting doctors who have participated in three clinical trials or sending doctors to participate in short-term (generally 3–6 months) clinical trial training courses held in high-level hospitals to gain the requisite experience. However, this has certain policy risks; for example, regulations generally require that the researchers participating in the project are generally employees of the unit (F.U.S.C. Center, 2021). Due to the development of clinical trials in low-level or private hospitals, the level of attention to this requirement is not very high. Therefore, very few hospitals will recruit a PI with experience in three clinical trial projects or send PIs from their own units to go out for further education to obtain the qualification.

Therefore, even though the application process has been greatly simplified, the number of hospitals in China that can conduct clinical trials has not exploded as anticipated. However, it has been an effective strategy to reduce supervision costs and thereby strengthen supervision. On 20 July 2022, the department of drug registration of NMPA issued a letter to strengthen the daily supervision of drug clinical trial institutions. There are indeed some hospitals in the letter that have been suspended from undertaking clinical trials due to PI qualifications (The letter has not been released to the public, and the relevant content can be queried in the record-keeping system. For example: institution name - Shanghai East Hospital, in the supervision and inspection information). This shows that although the regulations do not provide specific judicial interpretations for the three clinical trials, the regulatory authorities prefer multi-centre registered clinical trial projects that meet the higher requirements. This also reminds sponsors that when looking for new PIs and centres, it is best to investigate whether the PI has participated in three clinical trials and avoid regulatory risks that do not involve the authenticity of the experiment itself.

## Problems and challenges still facing

However, with the continuous updating of new treatment technologies, the supervision of clinical trials in China still needs further improvement. The requirements for researchers may need to be further strengthened, especially normative and scientific literacy requirements. It is foreseeable that due to the uneven clinical diagnosis and treatment level of a large number of new researchers (especially in grass-roots hospitals), the lack of standardization and the lack of full awareness of the risks brought about by new treatment techniques will inevitably have negative effects on clinical trial influences. This high demand for researchers in China is a strategy worthy of recognition. These alterations are all aimed at better protecting the rights of subjects and advancing the clinical trial process.

This is because PIs play a key role in clinical trials. And it is worth noting that the PI of clinical trials in China is usually the director of clinical departments. During the entire clinical trial period, the PI needs to be responsible for many things (Feehan and Garcia-Diaz, 2020), mainly including the following aspects: 1. Coordinate the parties involved in the clinical trial. 2. Reasonable division of labor and authorization for clinical trial staff. 3. Sign the research agreement and ensure that the department has sufficient conditions to carry out clinical trials. 4. Confirmation of some key reports and data. All these show that what an excellent PI needs is composite ability. In addition to clinical capabilities, PIs are also required to have excellent human resource management capabilities and coordination and communication skills (Foncubierta-Rodríguez et al., 2022). An excellent PI must be able to fully mobilize the enthusiasm of the research team, select suitable personnel (Foncubierta-Rodríguez et al., 2020), and conduct timely summary and review of clinical trials.

However, it is still worth noting that specific judicial interpretations should be made for the three clinical trials at the national level. Or provide some alternatives for evaluating the PI’s capabilities, because multi-dimensional evaluation of a PI’s ability is more advantageous (O'Kane et al., 2021). Especially to solve the difficulties of grass-roots hospitals and new PIs, and avoid being trapped in a chicken-and-egg cycle. This recommendation could be implemented by offering an alternative to designate PIs who, although they have not participated in three clinical trials, may demonstrate other types of competencies that support them as very suitable candidates for the development of these clinical trials. 1. The GCP exam is organized by the government, through the test scores to evaluate part of the PI’s ability; 2. Government recognized training mode, PI accumulates project experience in other hospitals; 3. Prove the clinical research ability of PI through a certain number of high-quality clinical research papers or projects of a PI; 4. PIs who do not meet the project experience are allowed to participate in phase III clinical trials. Because the risk to subjects of phase III clinical trials is generally low. All in all, regulatory authorities should evaluate the capabilities of PIs in multiple dimensions, so as to ensure the quality of clinical trials, better release clinical trial resources, and accelerate the development of new drugs.

## Data Availability

The original contributions presented in the study are included in the article/supplementary material, further inquiries can be directed to the corresponding authors.
